# The role of peripheral innate immune cells in Alzheimer’s disease progression

**DOI:** 10.3389/fimmu.2025.1616939

**Published:** 2025-07-16

**Authors:** Yanchun Cao, Ke Tang, Pengcheng Ma, Run Zhang, Yani Yang, Tingting Li, Ying Zhang, Xiaoming Peng

**Affiliations:** ^1^ School of Traditional Chinese and Western Medicine, Gansu University of Chinese Medicine, Lanzhou, Gansu, China; ^2^ Department of Neurology, Affiliated Hospital of Gansu University of Chinese Medicine, Lanzhou, Gansu, China; ^3^ Department of Psychiatry, The No.2 people’s hospital of Lanzhou, Lanzhou, Gansu, China; ^4^ Department of Psychiatry, Xiaogan City social welfare and medical rehabilitation center, Xiaogan, Hubei, China

**Keywords:** Alzheimer’s disease, peripheral innate immunity, neuroinflammation, trained immunity, genetic polymorphisms, myeloid-derived suppressor cells (MDSCs)

## Abstract

Alzheimer’s disease (AD) is a neurodegenerative disorder characterized by amyloid-β (Aβ) plaques, neurofibrillary tangles, and chronic neuroinflammation. While microglia and astrocytes dominate CNS immune responses, emerging evidence implicates peripheral innate immune cells (PIICs)—including neutrophils, monocytes, dendritic cells, NK cells, and myeloid-derived suppressor cells (MDSCs)—as critical modulators of AD pathogenesis. This review synthesizes recent advances linking PIIC-related genetic polymorphisms to AD susceptibility and progression. We highlight how PIICs traffic into the brain via chemokine signaling, where they exhibit stage-specific effects: early recruitment may limit Aβ deposition via phagocytosis, whereas chronic infiltration exacerbates neuroinflammation and neuronal death. Paradoxically, some PIICs exert immunosuppressive effects that could be harnessed therapeutically. We further discuss preclinical strategies to modulate PIIC function, such as CCR2 inhibition, neutrophil depletion, and MDSC adoptive transfer. By bridging peripheral and central immunity, this review unveils PIICs as promising targets for next-generation AD therapies, advocating for precision immunomodulation tailored to disease stages.

## 1 Introduction

Alzheimer’s disease (AD), a progressive neurodegenerative condition, is pathologically defined by the accumulation of extracellular Aβ plaques alongside intracellular neurofibrillary tangles composed of hyperphosphorylated tau protein (1). Although aging populations have intensified its global impact, current therapeutic strategies targeting conventional amyloid/tau paradigms show restricted efficacy (2). Emerging evidence indicates that AD pathogenesis encompasses diverse mechanisms, such as metabolic disturbances, impaired mitochondrial function, and particularly neuroinflammatory processes that initiate prior to observable pathology and propel disease advancement (3–5). Although transient neuroinflammatory responses serve protective functions, persistent inflammation worsens neuronal injury and hastens cognitive deterioration (6, 7), positioning it as a critical intervention point for halting the transition from mild cognitive impairment to AD (8).

Central nervous system (CNS) neuroinflammation is primarily regulated by microglia and astrocytes (9, 10), whereas PIICs contribute underrecognized yet vital functions. Diverging from traditional perceptions of innate immunity as nonspecific, PIICs—encompassing monocytes (PMCs), natural killer (NK) cells, and bone marrow progenitors—demonstrate adaptive characteristics through “trained immunity” enabling long-term immunological memory (11, 12). Their involvement manifests contrasting effects: beneficial immune monitoring during acute phases versus detrimental contributions in chronic AD progression (13). Contemporary findings reveal that PIICs (including polymorphonuclear neutrophils [PMNs], PMCs, dendritic cells [DCs], and NK cells) migrate into brain tissue, intensifying inflammatory cascades and neuronal damage (14–16). Chemotactic signals from peripheral Aβ recruit these cells, prompting secretion of proinflammatory factors and compromising blood-brain barrier integrity (17). While MDSCs have been extensively studied in oncology (18), their role in AD remains elusive. This review summarizes current knowledge on PIIC-associated genetic polymorphisms, quantitative and qualitative alterations in AD, and mechanistic insights into their CNS infiltration. Elucidating these processes may reveal innovative targets for pharmacological modulation in AD.

## 2 Peripheral innate immune in Alzheimer’s disease

### CD33

Advances in GWAS have consistently identified genetic variants influencing AD susceptibility, with a significant proportion localized to myeloid cell-specific genes—over one-third of risk loci exhibit myeloid-selective expression (19, 20). Epigenetic analyses further indicate that AD-associated GWAS loci are disproportionately enriched in enhancer regions regulating innate immune activity (21). Understanding how polymorphisms in PIIC-related receptors, cytokines, and complement factors contribute to AD is thus essential for unraveling disease mechanisms and developing targeted therapies. Expressed mainly on microglia and myeloid cells, the transmembrane receptor CD33 modulates intercellular adhesion and innate immune signaling (22). An AD-linked risk allele of CD33 correlates with increased receptor expression in affected brains, impairing microglial phagocytosis and Aβ42 clearance (23, 24). Initial evidence implicating CD33 emerged from family-based GWAS, which detected the rs3826656 variant within the gene’s 3′ region (25). Subsequent work by Bradshaw et al. (26) revealed elevated rs3865444-associated CD33 transcription in AD patients, with expression levels inversely correlated with cognitive performance. Mendelian randomization studies further support a causal role for CD33-dependent immune dysregulation in AD (27).

### 2.2 TREM family receptors in immune regulation

Of the innate immune markers associated with AD, TREM2 has garnered the most attention due to its dual-phase impact on disease progression: early protective effects via Aβ clearance followed by later detrimental neuroinflammatory responses (28–30). This membrane-bound receptor enhances phagocytic activity and modulates microglial function (31). The R47H variant of TREM2 elevates AD risk by 2–3-fold (32). Adjacent to TREM2 on chromosome 6p21.1, TREM1 is predominantly active in monocytes, macrophages, and neutrophils during immune challenges (33). In Han Chinese populations, the TREM1 SNP rs2062323 exhibits a protective effect, with the T allele significantly reducing AD susceptibility.

### 2.3Cytokine and complement in Alzheimer’s disease

Cytokines serve as pivotal mediators of PIIC-driven immunity. Genetic studies have associated polymorphisms in interleukin genes. Notably, carriers of risk alleles in IL1β, IL6, IL10, and TNF-αdisplay heightened susceptibility (34–37). While IL1α (rs1800587) and IL33 (rs11792633) correlate with late-onset AD in Han Chinese cohorts, IL1β lacks such an association (37). Complement system genes (CLU, CR1, SERPINA3, CFH, C4) also harbor AD-linked SNPs (38, 39). CLU, a major risk locus for late-onset AD, contains protective variants (rs11136000, rs2279590, rs9331888) that reduce Aβ accumulation (39, 40). Patients homozygous for the rs11136000-C allele exhibit the highest Aβ burden, whereas TT genotypes show minimal deposition (41). In APOE4-positive individuals, the C allele significantly elevates CSF tau levels, with CC homozygotes exceeding CT heterozygotes (42). The rs9331888-GG genotype further correlates with diminished hippocampal volume (41). Beyond common variants, rare mutations in PLCG2, ABCA7, SORL1, and ECE2 also modulate AD risk (43, 44). Collectively, PIIC-related genetic variants intricately influence AD pathogenesis, offering mechanistic insights and potential therapeutic avenues. Future studies must consider ethnic, geographic, and demographic variables when assessing these polymorphisms.

## 3 Peripheral innate immune cells in Alzheimer’s disease

### 3.1 Polymorphonuclear neutrophils

As the predominant myeloid cell type in human peripheral blood, neutrophils (PMNs) play crucial roles in maintaining tissue homeostasis while also contributing to inflammatory damage during sterile inflammation (45, 46). Research using AD mouse models reveals their early involvement in disease pathogenesis, with cerebral accumulation preceding clinical symptoms and subsequent release of pro-inflammatory factors (47, 48). A meta-analysis by Huang *etal.* demonstrated significantly elevated peripheral PMN counts in patients with mild cognitive impairment and AD compared with healthy controls, implicating oxidative stress, immune dysregulation and neuroinflammation in driving this expansion (49). Through enhanced antigen presentation, these cells stimulate T lymphocyte activation, creating a feedback loop that amplifies TNF-α production (50).

**Figure 1:**
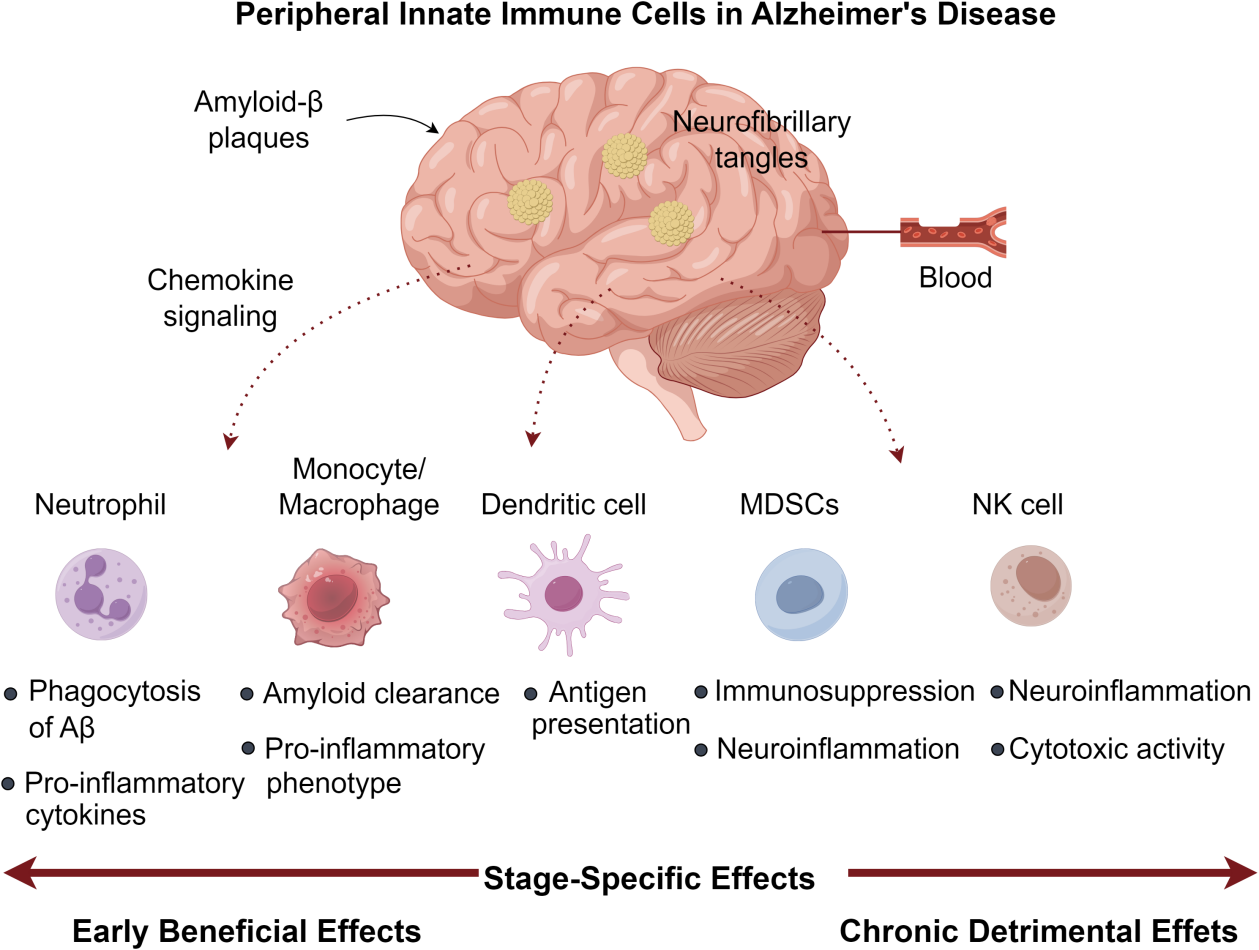
Peripheral innate immune cells in Alzheimer’s disease.

Chronic TNF-α modulation in 3×Tg AD mice demonstrated dual effects: promoting PMN infiltration (CD45Hi/CD11b^+^/GR1^+^/1A8^+^) alongside Aβ/tau accumulation, yet paradoxically enhancing memory function (51). The neurotoxic potential of activated PMNs stems from myeloperoxidase (MPO), ROS, and NET generation - all capable of compromising blood-brain barrier integrity (52, 53), with circulating MPO levels predicting cognitive deterioration (54). Experimental PMN depletion during early-stage AD yields lasting cognitive improvements in aged models, highlighting their disease-modifying capacity (47, 55). Clinical studies consistently associate elevated peripheral PNLR values with AD progression markers, including CSF Aβ reduction, tau elevation, and hippocampal volume loss (15, 50). This ratio may signify dysregulated immune responses correlating with amyloid accumulation and progressive cognitive impairment (15, 56). Mechanistic studies employing microfluidic AD models reveal Aβ-stimulated microglia secrete IL-6, IL-8, and CCL2, with cytokine neutralization preventing PMN CNS migration (57). Aβ_42_ further potentiates neuroinvasion by modulating LFA-1 affinity states, increasing endothelial ICAM-1 binding (47). Pharmacological LFA-1 inhibition attenuates PMN recruitment, ameliorates neuropathology, and restores cognitive function in AD mice (47, 58). Intracerebral PMNs localize to amyloid deposits, where MPO, NETs, and IL-17 perpetuate neuroinflammation (47). Their interactions with microglia induce MIF and IL-2 secretion (57), while the latter reduces plaque load and enhances synaptic function (59, 60). Thus, PMNs demonstrate pleiotropic neuroprotective and neurotoxic effects in AD pathogenesis (**Figure 1**).

### 3.2 Peripheral monocytes/macrophages

Mononuclear phagocytes (PMCs) exhibit remarkable functional plasticity, dynamically responding to pathological stimuli through subset-specific adaptations. Human monocyte populations are classified based on surface markers into three distinct subsets: classical (CD14^+^CD16^+^), intermediate (CD14^+^CD16^+^), and non-classical (CD14^dim^CD16^+^). In Alzheimer’s disease (AD), shifts in monocyte distribution occur, characterized by increased non-classical and intermediate subsets alongside decreased classical monocytes (61). During neuroinflammatory conditions, classical monocytes preferentially infiltrate the CNS, where microglial activation by Aβ upregulates CCR2 chemokines, facilitating monocyte recruitment, macrophage differentiation, and amyloid clearance (62). Genetic ablation of CCR2 in AD models reduces cerebral PMC numbers (63), whereas pharmacological CCR2 blockade exacerbates amyloid accumulation and cognitive decline (64). Clinical studies reveal diminished monocytic CCR2 expression but elevated circulating CCL2 in AD patients, indicating impaired CCR2-CCL2 signaling and defective migration (65).

The mitochondrial translocator protein (TSPO), highly expressed in AD-associated microglia (66), has recently been identified as a regulator of PMC chemotaxis, with TSPO inhibitors suppressing Aβ-induced monocyte migration (67). Despite entering the CNS, PMCs demonstrate impaired phagocytic activity, leading to neuronal damage (68). Age-dependent reductions in Aβ_42_ uptake across all monocyte subsets are more pronounced in AD, as reported by Chen et al. (69), potentially due to decreased Toll-like receptor 2 (TLR2) expression, which compromises Aβ recognition. Furthermore, PMCs adopt a pro-inflammatory state, releasing elevated levels of cytokines (IL-6, IL-1β, TNF) and inflammasome components (NLRP3, IL-18), alongside increased HLA-DRA expression, thereby exacerbating neurodegeneration (61). Notably, PMC transcriptional profiles shift during disease progression, with anti-inflammatory IL-10 predominating in early-stage AD before transitioning to a sustained pro-inflammatory signature (61), suggesting temporally distinct roles—neuroprotective in prodromal phases but detrimental in later stages. Overexpression of ACE in CD115^+^ PMCs has been shown to mitigate neuropathology and improve cognition in APP^RE^/PS1 transgenic mice (70). Emerging therapeutic strategies may involve bone marrow-derived progenitor cell transplantation to restore functional PMC populations (32) (**Table 1**).

**Table 1:** Peripheral Innate Immune Cells (PIICs) in Alzheimer’s disease: mechanisms and therapeutic implications.

Cell type	CNS infiltration mechanisms	Stage-specific roles	Therapeutic strategies
Polymorphonuclear Neutrophils (PMNs)	CCR2/CCL2 axis; LFA-1/ICAM-1 binding; Aβ-induced IL-8 secretion	**Early**: Phagocytic Aβ clearance; **Late**: NETosis, ROS/MPO-mediated neurotoxicity	LFA-1 inhibitors (Lifitegrast); MPO inhibitors; CXCR2 antagonists (Reparixin)
Monocytes/Macrophages	CCR2/CCL2 chemotaxis; TSPO-dependent migration	**Early**: Aβ phagocytosis; **Late**: Pro-inflammatory (IL-1β, TNF-α) polarization	CCR2 antagonists (PF-04178903); TSPO modulators; ACE-overexpressing cell therapy
Dendritic Cells (DCs)	Potential CCL19/CCL21-mediated migration (unconfirmed)	**Early**: Antigen presentation; **Late**: Dysregulated cytokine production	DC-based Aβ vaccines; PD-L1/IL-12 pathway modulation
Natural Killer (NK) Cells	CX3CL1/CX3CR1 axis; CCR7 dysfunction in MCI/AD	**Early**: Viral surveillance; **Late**: IFN-γ-driven microglial activation	NK cell depletion (anti-NK1.1); CX3CR1 blockade; IFN-γ neutralization
Myeloid-Derived Suppressor Cells (MDSCs)	HIF-1α/MIF pathway; CCL2/CXCL8 chemotaxis	**Early**: Immunosuppression (IL-10/TGF-β); **Late**: Functional exhaustion	M-MDSC adoptive transfer; MIF inhibitors (Ibudilast)

### 3.3 Dendritic cells

Dendritic cells (DCs) serve as critical intermediaries between innate and adaptive immune responses. As these antigen-presenting cells undergo maturation, their migration from circulation to peripheral tissues results in decreased blood DC populations (71). While definitive evidence of peripheral DC infiltration into the AD brain remains elusive, studies in APP/PS1 transgenic mice demonstrate that systemic DC depletion leads to increased amyloid plaque burden, suggesting their potential role in cerebral amyloid clearance (72). This hypothesis is supported by clinical observations of decreased circulating DC precursors (73) and myeloid DC subsets (74) in AD patients, consistent with possible CNS migration. The causal relationship between DC reduction and neurodegenerative processes requires further clarification.

Research by Ciaramella et al. (74) identified an association between lower myeloid DC counts and both disease advancement and depressive symptoms in AD. In contrast, 5×FAD transgenic models exhibit altered DC function characterized by increased IL-12 and MIP-1 production in mesenteric lymph node myeloid DCs, coupled with decreased PD-L1 expression, indicating potential DC dysfunction in AD (75) (**Table 1**). Current understanding of DC involvement in AD remains limited, with particularly scarce data regarding DC-based immunization strategies. One study demonstrated cognitive improvement in APPswe/PSEN1ΔE9 mice following co-immunization with Aβ_4_-pulsed DCs and splenocytes (76). DC-based immunization approaches may offer advantages over conventional protein vaccines by simultaneously engaging both innate and adaptive immunity to generate targeted antibody responses and enhance pathogen clearance. However, clinical translation requires thorough investigation of multiple parameters including DC development, phenotypic characteristics, proliferative potential, antigen recognition profiles, post-activation behavior, and safety considerations (71).

### 3.4 Natural killer cells

Natural killer (NK) cells, a specialized group of innate immune lymphocytes, are increasingly recognized as key modulators of neuroinflammatory processes (77). These cytotoxic lymphocytes are primarily categorized into two functionally distinct populations based on CD56 expression levels: the relatively scarce CD56bright subset that specializes in cytokine production, and the more prevalent CD56dim population that mediates cell-killing activity (77). Current understanding of NK cell characteristics in Alzheimer’s pathology reveals conflicting observations. While some investigations report no significant differences in NK cell numbers or functional capacity between AD patients and healthy individuals (78), other studies demonstrate decreased peripheral NK cell counts with concurrent upregulation of immune-related genes (79). Research by Qi et al. (77) revealed a reduction in both NK cell numbers and cytotoxic potential in AD patients, along with identification of an expanded CX3CR1^+^TBX21^+^ NK subpopulation showing an inverse correlation with cognitive scores. Conversely, Solerte et al. (80) reported enhanced cytotoxic activity and increased TNF-α/IFN-γ production in AD-derived NK cells compared to age-matched controls, similarly associating with poorer cognitive outcomes. Experimental NK cell elimination in 3×Tg AD mice was shown to reduce neuroinflammation and cognitive deficits while preserving neural progenitor populations, though without affecting amyloid deposition (81).

The functional state of NK cells is determined by the integration of signals from both stimulatory and inhibitory surface receptors. Analysis of receptor expression patterns revealed preserved levels of CD57, NKG2D and CD94 in mild cognitive impairment (MCI) and early AD patients relative to healthy elderly subjects, while NKG2A showed selective reduction in MCI cases, a change that may promote NK cell activation (82). These partially contradictory findings collectively suggest that while NK cell alterations occur in AD, their exact role in disease mechanisms remains unclear. Comprehensive investigations employing single-cell transcriptomics coupled with advanced immunophenotyping are needed to fully characterize NK cell diversity across different AD stages. Although NK cell presence has been confirmed in AD animal models, their involvement in human disease requires further validation (83). Glial cells (microglia and astrocytes) contribute to NK cell recruitment and sustained neuroimmune activation through cytokine and chemokine secretion (84). NK cell-derived IFN-γ can polarize microglia toward a pro-inflammatory state, resulting in suppressed hippocampal neurogenesis and the development of cognitive deficits and depressive-like symptoms (85). Distinct NK cell subsets exhibit differential chemokine receptor expression patterns: while CX3CR1 facilitates CD56dimCD16^+^ NK cell brain infiltration in multiple sclerosis (86), its expression remains unaltered on NK cells from MCI and AD patients (82). Interestingly, CCR7 (the receptor for CCL19/CCL21) shows increased expression in MCI-derived NK cells, yet their migratory response to CCL19 is impaired in both MCI and AD cases (82) (**Table 1**).

### 3.5 Myeloid-derived suppressor cells

Myeloid-derived suppressor cells (MDSCs) represent a heterogeneous population of immature myeloid precursors that emerge in response to inflammatory signals. These immunoregulatory cells are broadly classified into monocytic (M-MDSCs) and granulocytic (PMN-MDSCs) subtypes, both capable of suppressing effector immune cell functions through potent inhibition of mature myeloid and lymphocyte populations (87). Under chronic inflammatory conditions, MDSCs migrate to affected tissues where they help regulate immune responses and prevent excessive activation. In Alzheimer’s disease, available evidence suggests a dynamic pattern of MDSC involvement, with expansion during initial disease phases followed by progressive decline (88). Clinical studies reveal distinct alterations in MDSC populations across disease stages. Le Page et al. (89) observed increased circulating CD33^+^HLA^-^DR^-^ M-MDSCs and CD33^+^HLA^-^DR^-^CD11b^+^CD15^+^ MDSCs in mild cognitive impairment (MCI) patients compared to both AD patients and healthy controls. Similarly, Thome et al. (61) reported enhanced MDSC frequency and immunosuppressive activity in early AD, associated with reduced pro-inflammatory gene expression in peripheral blood mononuclear cells - effects that diminished with disease progression (**Table 1**).

The neuroprotective potential of MDSCs stems from their ability to secrete immunomodulatory cytokines that promote microglial polarization toward an anti-inflammatory M2 phenotype. While this shift may help mitigate neuroinflammation and neuronal damage, it could simultaneously impair clearance of pathological protein aggregates (90). The temporal pattern of MDSC dynamics suggests an endogenous regulatory mechanism that becomes inadequate as AD advances (88). This observation has spurred interest in therapeutic strategies aimed at sustaining MDSC-mediated immunosuppression, as demonstrated by studies showing that M-MDSC transplantation can counteract immune dysregulation and cognitive deficits in AD mouse models (91). Despite these findings, direct evidence of MDSC infiltration in the AD brain remains elusive. Potential mechanisms for CNS recruitment may involve the HIF-1α/MIF pathway, known to mediate MDSC trafficking in cancer (92), given the elevated cerebrospinal fluid MIF levels observed in AD patients (93). Additionally, various chemokines upregulated in AD may facilitate MDSC migration toward affected brain regions (90). Further research is needed to clarify the spatial and temporal distribution of MDSCs in AD pathogenesis and their potential as therapeutic targets (**Figure 1**).

## 4 Therapeutic landscape

Despite decades of research, therapeutic options for AD remain limited, with most treatments offering only symptomatic relief rather than disease modification (94). Recently, however, the therapeutic landscape has evolved to include several disease-modifying treatments (DMTs), particularly monoclonal antibodies that target Aβ (95). For example, aducanumab and lecanemab, two anti-Aβ monoclonal antibodies aim to reduce Aβ plaque burden through immunotherapeutic mechanisms (96). Aducanumab selectively binds to aggregated Aβ and facilitates its clearance, while lecanemab preferentially targets soluble Aβ protofibrils, mitigating their synaptotoxic effects (97). Other experimental approaches include tau-targeting immunotherapies, such as semorinemab, that aim to neutralize pathological tau species and interrupt their propagation between neurons (98). However, these therapies have shown limited benefits in clinical trials, and cause adverse effects such as amyloid-related imaging abnormalities-edema (99, 100). Compared to these approaches, immunomodulatory strategies targeting peripheral innate immune cells represent a novel paradigm. These include DC-based immunization approaches and CCR2 regulator to facilitate monocyte infiltration and macrophage differentiation to restore immune homeostasis. While still in preclinical stages, these therapies offer the advantage of modulating upstream inflammatory signals that orchestrate central pathology. Thus, contextualizing these immunotherapies within the current AD brain immune landscape not only clarifies their unique positioning but also highlights the need for stage-specific, precision immunomodulation that integrates both central and peripheral immune mechanisms.

## 5 Conclusion

Recent advances have revealed that PIICs are not mere bystanders but active participants in Alzheimer’s disease (AD) progression. This review highlights the stage-specific and context-dependent roles of PIIC subsets, such as neutrophils, monocytes/macrophages, dendritic cells, NK cells, and MDSCs, in shaping the neuroinflammatory landscape of AD. Early recruitment of PIICs may aid in Aβ clearance and immunoregulation, while persistent activation exacerbates neurotoxicity via cytokine storms, oxidative stress, and blood–brain barrier disruption. Genetic polymorphisms in PIIC-associated genes, such as CD33 and TREM2, further underscore their mechanistic relevance and suggest population-specific susceptibilities.

Targeting innate immune pathways offers a promising yet complex therapeutic avenue. Modulation of chemokine receptors, cytokine secretion, and macrophage function has shown efficacy in preclinical models. However, challenges remain, including the heterogeneity of immune responses across AD stages, potential off-target effects, and limited understanding of long-term immunomodulation. Future drug development should prioritize precision strategies that harness the protective capacity of PIICs while minimizing chronic inflammation. Integrating multi-omics profiling, advanced immunophenotyping, and longitudinal clinical data will be essential to refine these approaches. Ultimately, a deeper understanding of innate immunity may unlock novel interventions capable of altering AD trajectory in its earliest and most modifiable phases.
